# Use of geographic information system tools to predict animal breed suitability for different agro-ecological zones

**DOI:** 10.1017/S1751731118003002

**Published:** 2018-11-13

**Authors:** M. Lozano-Jaramillo, J. W. M. Bastiaansen, T. Dessie, H. Komen

**Affiliations:** 1 Wageningen University & Research Animal Breeding and Genomics, P.O. Box 338, 6700 AH Wageningen, The Netherlands; 2 International Livestock Research Institute, P.O. Box 5689 Addis Ababa, Ethiopia

**Keywords:** agro-ecology, breeding programs, distribution models, livestock, local adaptation

## Abstract

Predicting breed-specific environmental suitability has been problematic in livestock production. Native breeds have low productivity but are thought to be more robust to perform under local conditions than exotic breeds. Attempts to introduce genetically improved exotic breeds are generally unsuccessful, mainly due to the antagonistic environmental conditions. Knowledge of the environmental conditions that are shaping the breed would be needed to determine its suitability to different locations. Here, we present a methodology to predict the suitability of breeds for different agro-ecological zones using Geographic Information Systems tools and predictive habitat distribution models. This methodology was tested on the current distribution of two introduced chicken breeds in Ethiopia: the Koekoek, originally from South Africa, and the Fayoumi, originally from Egypt. Cross-validation results show this methodology to be effective in predicting breed suitability for specific environmental conditions. Furthermore, the model predicts suitable areas of the country where the breeds could be introduced. The specific climatic parameters that explained the potential distribution of each of the breeds were similar to the environment from which the breeds originated. This novel methodology finds application in livestock programs, allowing for a more informed decision when designing breeding programs and introduction programs, and increases our understanding of the role of the environment in livestock productivity.

## Implications

Understanding the environmental requirements of different breeds, including the knowledge of which environmental variables are determining the difference in performance, is an important tool to support higher productivity in particular regions. Having this information will help us make predictions of where different breeds can be more productive and where breeding and introduction programs can be performed. The results of this research suggest the use of the presented methodology that uses habitat distribution models to be able to predict breed suitability in introduction programs or testing schemes.

## Introduction

Indigenous breeds are exposed to natural selection processes that allow them to acquire qualities that make them better suited to their environment. Native breeds have been described to be locally adapted to specific environmental conditions, as well as tolerant to different parasites and diseases (Solti *et al*., 2000; Köhler-Rollefson *et al*., 2009; Mirkena *et al*., 2010). Exotic breeds show an advantage in production over the indigenous breeds as they have been selected for high productivity for many generations. For this reason, many introduction programs aim to increase local egg and meat productivity in chicken, to increase wool yield in sheep, meat quality in cattle and in goats, as well as milk yield in cows. However, most programs were not successful, mainly because of the non-adaptability of the exotic breeds to the challenging tropical environments (Kosgey *et al*., 2006; Mirkena *et al*., 2010; Wurzinger *et al*., 2011; Haftu Kebede, 2016). What are needed are methods to predict which areas are suitable, in terms of environmental conditions, for the introduction of different breeds. Such methodology would make introduction programs and design of breeding programs more efficient.

Predictive habitat distribution models are Geographic Information Systems (GIS)-based tools that use the current climatic conditions of a species to make predictions of the potential distribution of the species (Pearson and Dawson, 2003; Hijmans and Graham, 2006; Soberón and Nakamura, 2009). These tools explain naturally occurring plant and animal distribution patterns, or assess the impact of climate change on their distributions. These models may be useful as a new tool to predict whether the introduction of a specific livestock breed has the potential to be successful, based on local climatic conditions. In livestock research, GIS tools have been used to map suitable territories for land use (Malafant, 1998; Kalivas and Apostolopoulos, 2005), to view patterns of disease transmission (Cringoli *et al*., 2007) and to establish conservation priorities (Bertaglia *et al*., 2007). However, these tools have not been used to predict suitable areas for particular livestock breeds. Neither have they been used to understand the environmental factors that may influence changes in productivity between environments.

Here we present a methodology that uses GIS tools to develop predictive habitat distribution models that can be used to predict the suitability of a breed for a particular region based on climatic information. The methodology was tested on two introduced poultry breeds in Ethiopia. Ethiopia was considered suitable for testing the methodology because it is an ecologically diverse country with a broad range of contrasting agro-ecologies defined by altitude, temperature and rainfall (Mengistu, 2003). The methodology was used to (1) make predictions on the potential suitable habitat range for each breed; (2) indicate which bio-climatic and land cover variables explain the differences between the areas predicted to be suitable for the different breeds; and (3) establish a ranking of the suitability of the two available breeds for each region. This novel methodology finds application in livestock programs, allowing a more informed decision making for the design of breeding programs and introduction programs, and increases our understanding of the role of the environment in livestock productivity.

## Material and methods

Using distribution models and GIS tools we developed a methodology and applied it to predict areas of potential suitability for two different livestock breeds. To validate the methodology we chose two different chicken breeds that are currently kept in Ethiopia. The development of the methodology involved building distribution models based on climate for each breed. Validation was done by cross-validation to determine if the model could differentiate areas where the breeds are kept from areas where the breed is not present.

### Distribution model building

To build the distribution models, we used the maximum entropy algorithm implemented by Maxent (Phillips *et al*., 2006). Maxent is one of the most commonly used tools in ecology to predict species distributions. It has been shown to have greater predictive power than other tools, particularly for small data sets (Elith *et al*., 2006). Maxent is a machine learning algorithm that uses presence-only data to relate the environmental variables and occurrence points to establish a probability of potential geographic suitability (Phillips *et al*., 2006; Phillips and Dudík, 2008). The output, is the probability of suitability for all map positions that can be represented as a heat map.

### Environmental data

**Figure 1:**
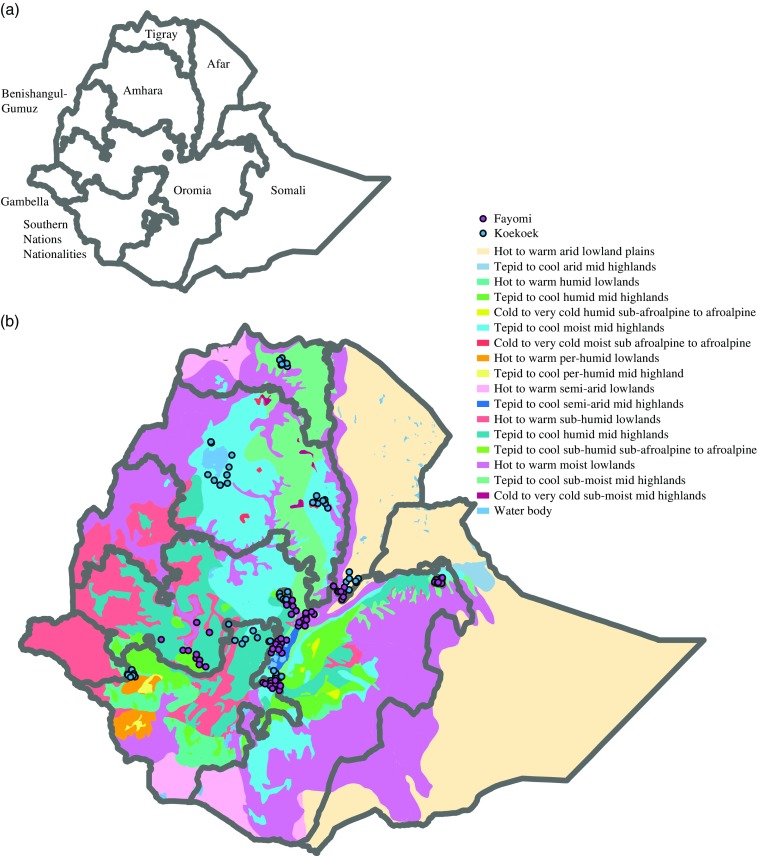
Map of Ethiopia showing (a) its nine regional states, and (b) the 18 major agro-ecological zones based on temperature and precipitation. Dots on the maps indicate the localities from each of the breeds that were used to build the models.

Ethiopia was chosen because it is a diverse country divided in nine regional states (Figure 1a) and five agro-ecological zones based on rainfall and elevation (Table 1), the latter being a determinant for agricultural land use due to its influence on temperature (Mengistu, 2003; Deressa *et al*., 2010). In addition, to better characterize the country’s temperature and moisture regimes, a system of zonation was developed generating 18 major zones (Deressa *et al*., 2010; Figure 1b).

**Table 1:** Traditional agro-ecological zones in Ethiopia

Zone	Elevation (m)	Mean annual precipitation (mm)	Average annual temperature (°C)
Bereha (dry-hot/desert)	<500	<200	>27.5
Kola (sub-moist warm/lowlands)	500 to 1500	200 to 800	20.0 to 27.5
Weinadega (dry-warm/mid-highlands)	1500 to 2500	800 to 1200	17.0 to 20.0
Dega (cold/highlands)	2500 to 3500	900 to 1200	11.5 to 17.0
Wurch (very cold or alpine/upper highlands)	>3500	900 to 2200	<11.5

Chickens are part of the Ethiopian village production systems, where they rely on scavenging for survival. Food source is dependent on seasonality, which in Ethiopian agricultural circumstances is strongly related to temperature and rainfall. We chose the sets of environmental variables that would represent trends in seasonality (temperature and precipitation); variables that would have an influence on, or would reflect on the adaptability, hence the biology of the chickens. A total of 21 variables (Supplementary Table S1) available at a 1 km by 1-km resolution were collected from WorldClim (Hijmans *et al*., 2005: http://www.worldclim.org/), and the Harmonized World Soil Database v 1.2 (Food and Agriculture Organization (FAO) *et al*., 2012). The environmental data included 19 bioclimatic variables and an elevation layer representing current climatic conditions. These 20 layers are commonly used as indicators of annual trends in seasonality, temperature and precipitation. In addition, a land cover layer, total cultivated land, was included as a proxy to anthropogenic intervention and agricultural systems, as smallholders occurrence and poultry density are closely linked in Ethiopia (Dessie, 2003; Mwacharo *et al*., 2013).

These environmental variables can be correlated. However, to avoid overfitting we used Maxent, which uses a regularization parameter to smoothen the model. It will reduce the importance of variables in the model when they are either of low predictive value or highly correlated to other variables (Phillips *et al*., 2006). Using this regularization parameter has been shown to perform better than other procedures that use other modeling methods to pre-select variables (Elith and Leathwick, 2009; Elith *et al*., 2011).

### Poultry production system in Ethiopia

Poultry production in Ethiopia is dominated by smallholder producers where nearly all rural and peri-urban families keep a flock of free range chickens in a scavenging system (Moges *et al*., 2010; Ravindran, 2010). Village production systems (also denoted traditional or backyard) account for 97% of the poultry production in Ethiopia, making the productivity highly dependent on the environment. In these systems chickens rely almost entirely on scavenging for feed. The amount of nutrients available depends on the region and season of the year. Between rainy seasons feed is limited because the land where chickens usually scavenge is used to grow crops. Attempts to improve the poultry sector in the country have been done through the introduction of exotic chicken breeds, but with no emphasis on changing the husbandry practices. Therefore, exotic breeds are kept under the same backyard conditions as the indigenous chickens (Habte *et al*., 2013).

### Breeds and occurrence

Two exotic breeds were selected for this study based on prior knowledge about their presence in smallholder farms in Ethiopia. The Fayoumi breed originates in Egypt and is said to be adapted to hot and very dry areas in tropical and sub-tropical conditions (Geleta *et al*., 2013). The Koekoek breed, developed in South Africa, is popular among South African farmers, and said to be adapted to the local conditions in South Africa (Grobbelaar *et al*., 2010). For Ethiopia, a total of 161 breed locations were used, 62 for the Fayoumi breed and 99 for the Koekoek breed (Figure 1b). These locations were obtained from the National Research Institute that handles the poultry database in the country; the Ethiopian Institute of Agricultural Research (EIAR).

### Predicting breed occurrence

Using the environmental variables selected previously, for each breed independently, we generated a map of the potential distribution given the current climatic and land cover conditions. The range of the potential distributions of both breeds was visualized and assessed in a heat map of the country. To distinguish climatically suitable from unsuitable areas, we applied the ‘minimum training presence’ threshold rule which uses the least suitable training occurrence record as the threshold (Pearson *et al*., 2007; Norris, 2014). Following the map generation, we validated the model using the receiver operating characteristic (ROC) curve and a binomial test of omission (known areas of presence predicted absent, Phillips *et al*., 2006). The ROC analysis is a standard approach to test model performance by evaluating the sensitivity (absence of omission error) and 1-specificity (commission error). For each breed the environmental variables that had the highest predictive contribution while building the model were identified.

### Cross-validation

To determine if the model predictions could predict breed suitability, we first divided the country in 1×1 decimal degree grids, which gave us a total of 110 cells. The grid was applied to limit the effect of spatial clustering on the cross-validation. For each breed independently, instead of removing points one by one, all the localities within each cell where the breed was present were removed from the training data set. This was done cell by cell for all of the cells that included the occurrence data. Once the occurrence points were removed from the cell, the model was fitted to predict a probability of occurrence for that same cell.

For the cells where the breed was not present, a set of random locations were defined as absent. This set of absent locations was created using ArcGIS v10.3.1. For each of these cells, the set of random localities were removed from the training data set, and then the model was fitted to estimate the mean predicted probability for each of the cells where the localities were removed. This was done cell by cell for all of the cells with the absence localities. Similar validation designs (variations on the *k*-fold cross-validation) are used for other approaches in wildlife species to develop more rigorous ecological niche models (Muscarella *et al*., 2014).

To base our results on suitable environmental conditions where poultry exists in Ethiopia, we first selected the cells with reported poultry density greater than 10 individuals/km^2^ (Robinson *et al*., 2014). Then from those cells we selected the ones where each breed was present, hence 78 of the 110 total cells for Fayoumi, and 76 cells of the total 110 cells for Koekoek were included, and the predicted probabilities for the cells with occurrences and the cells with absences were compared. The difference in probabilities between cells with occurrence and with absence was visualized with a density plot; a *t*-test was applied to test whether both groups were significantly different. An R-script implementing the cross-validation is included (Supplementary Material S1).

### Breed ranking

For each of the nine regional states in Ethiopia, we established the percentage of area predicted as suitable. Using ArcGIS v10.3.1 we calculated the total area per region, and the total area predicted as suitable for each of the breeds in each region. Finally for each breed we ranked the five regions that had the highest percentage of potential suitable area.

Data handling and Maxent algorithm (Phillips *et al*., 2006) which was implemented using the dismo package (Hijmans *et al*., 2017), were conducted with R version 3.2.2 (R Development Core Team, 2016) running on RStudio version 0.99.902 (RStudio Team, 2015).

## Results

### Prediction and ranking of suitability for breeds

**Figure 2:**
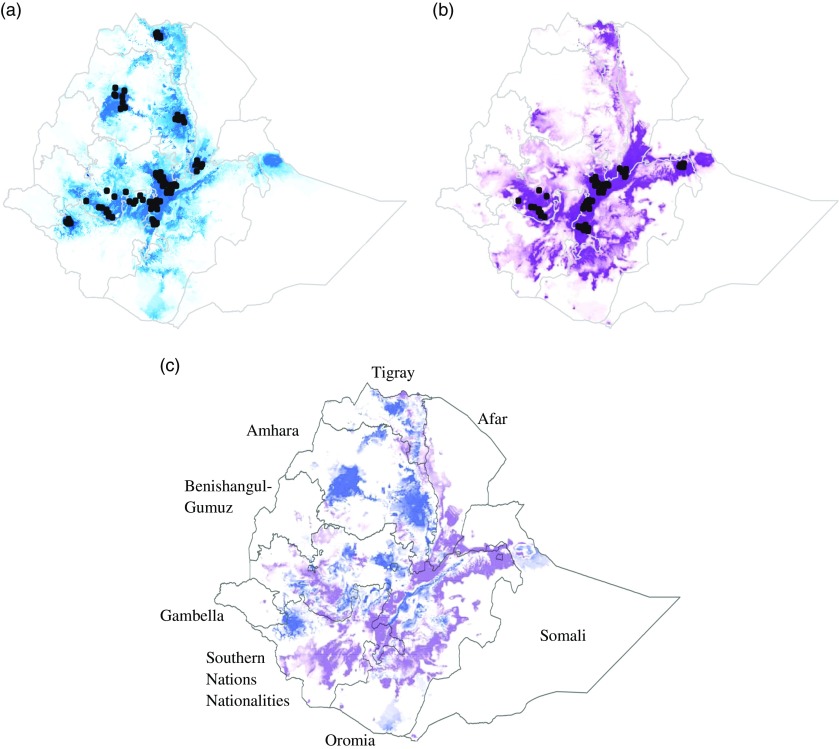
Suitability predictions for (a) Koekoek, and (b) Fayoumi chicken breeds in Ethiopia. Predicted areas are shaded; darker colors denote areas of higher climatic suitability. Observed localities used to build the model are shown in black dots. Ratio of suitability between chicken breeds (c). Purple color indicate higher predicted suitability for Fayoumi than for Koekoek. Blue color indicate higher predicted suitability for Koekoek than for Fayoumi.

For both breeds, the model predicted that suitable environmental conditions exist beyond the current distribution of the breed (Figures 2a and 2b). The area under the ROC curve for the model predicting the potential distributions of the Fayoumi and Koekoek breeds was close to one (0.981 and 0.975, respectively), indicating that the model performed well.

The percentage of area predicted as suitable for each of the nine regional states in Ethiopia differed between breeds (Figure 2c). For the Fayoumi breed, the four regional states with highest percentage of area predicted as suitable were Oromia, Southern Nations Nationalities and People’s Region (SNNPR), Amhara and Tigray (10.9%, 9.13%, 1.29% and 0.57%, respectively; Table 2). For the Koekoek breed the four regional states with highest percentage of area predicted as suitable were Amhara, Oromia, SNNPR and Tigray (12.93%, 10.41%, 9.45% and 0.74%, respectively; Table 2).

**Table 2:** Percentage of area predicted as suitable in the top four regions for each chicken breed

Chicken breed	Region	Percentage of area predicted as suitable
Koekoek	Amhara	12.93
	Oromia	10.41
	SNNPR	9.45
	Tigray	0.74
Fayoumi	Oromia	10.9
	SNNPR	9.13
	Amhara	1.29
	Tigray	0.57

SNNPR=Southern Nations Nationalities and People’s Region.

### Most important climatic conditions

Differences in habitat suitability were supported by differences in environmental conditions (Supplementary Figure S1). The variable explaining most of the variation in suitability for the Fayoumi breed (43.7%; Table 2) was associated to total cultivated land. The next two axes (jointly accounting for 26.3% of the environmental variation; Table 3) were associated to precipitation. For the Koekoek breed, the variable explaining most of the variation (PC1; 18.1%; Table 3) was the minimum temperature of the coldest month, and the next two axes (jointly accounting for 21.6% of the variation; Table 3) were associated to mean temperature of the warmest quarter, and the range of mean monthly temperature.

**Table 3:** Selected environmental variables with their percent contributions to the prediction for each chicken breeds’ model using Maxent

Chicken breed	Environmental variable	Percentage contribution
Koekoek	Min temperature of coldest month	18.1
	Mean temperature of warmest quarter	11.9
	Mean diurnal range	9.7
Fayoumi	Total cultivated land	43.7
	Precipitation of driest quarter	16.9
	Precipitation of coldest quarter	9.4

### Cross-validation

**Figure 3:**
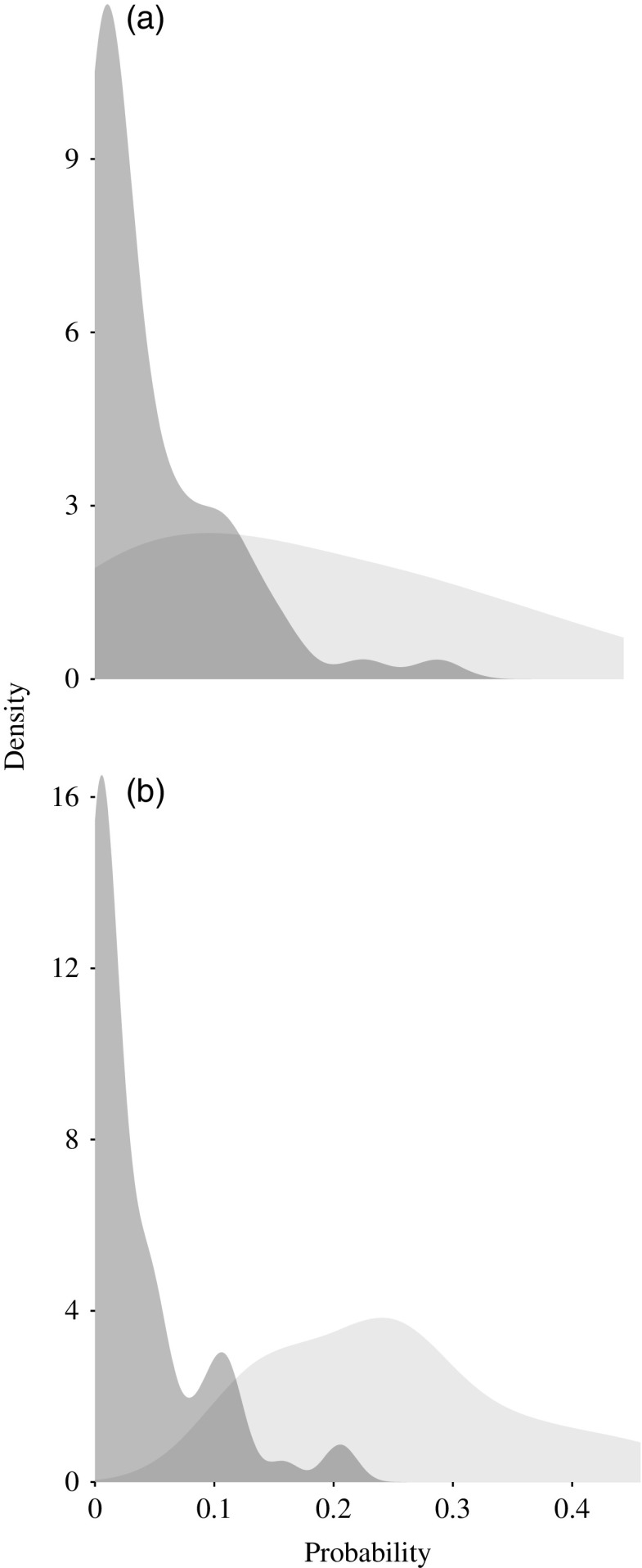
Density plots showing the probability predicted as suitable for the cells where the (a) Koekoek and (b) Fayoumi chicken breeds occur (in light grey) and where they are absent (dark grey).

For each of the breeds the mean predicted suitability for the occurrence cells was greater than the mean predicted suitability for the absence cells (*P*<0.05). For the Koekoek breed, the mean predicted suitability for the cells with absences was 0.047, and for the cells with occurrences was 0.167. For the Fayoumi breed, the mean predicted suitability for the absences was 0.036 and 0.249 for the occurrences (Figure 3).

## Discussion

A variety of GIS-based tools have been applied in agriculture. In goats and sheep they have been used to characterize their production system (Malafant, 1998), to propose pasture areas in regions where land has been fragmented (Kalivas and Apostolopoulos, 2005), and to analyze the spatial link between indigenous breeds and areas of livestock usage (Bertaglia *et al*., 2007). In cattle, buffaloes and sheep, GIS has been applied to see the spatial structure of animal populations, and to evaluate the characteristics of disease transmission between farms (Cringoli *et al*., 2007). In domestic fowl species, GIS was used to examine the extent of the ecological tolerance of an ancestor bird species to evaluate the success of domestication (Pitt *et al*., 2016). However, the use of habitat prediction models based on climate and land cover, have not been applied to an animal-breeding context. Here we show how these tools that are widely applied in wild species to cover diverse topics in biogeography, conservation and climate change, can be applied to in livestock to predict breed-specific environmental suitability.

Our results suggest that the two breeds that were tested occupy different climatic environments; the Fayoumi breed is suitable for areas where there is a higher percentage of land used in agriculture, and where there is higher precipitation, whereas the Koekoek breed is suitable in colder environments with larger temperature fluctuation. Even though in our dataset the breeds were kept in overlapping areas of the country, they do not always occur together. The Koekoek breed is kept in some localities with tepid to cool moist and sub-moist mid-highlands. The Fayoumi breed is kept in tepid to cool humid mid-highlands, and hot to warm moist lowlands. Temperature and rainfall were found to be the main drivers of the differences in the potential distribution. These climatic parameters are likely to affect livestock production and are highly distinctive between the agro-ecologies within our data set.

The distribution models indicated that the suitable areas for both of the breeds extend beyond their current boundaries, which suggests that there are more areas of the country where the breeds could be suitable for poultry production. The model was sensitive enough to distinguish between breeds. Areas that were predicted as highly suitable differed between the breeds were found to have significant climatic differences. For the Koekoek breed, the model predicted higher suitable cooler areas in the northern and southern parts of Ethiopia, whereas for the Fayoumi breed, humid areas toward the center of the country were predicted as highly suitable. Knowledge on the environmental conditions that can have an effect on the breeds’ performance is of crucial importance when deciding where to introduce them and where to maintain them.

Adaptability to different environments can be explained by looking at the breeds’ origin, where environmental and anthropogenic selective pressures have shaped their adaptation to specific environments. The Fayoumi is a breed of Egyptian origin (Hossaryl and Galal, 1994), while the Koekoek originated in South Africa (Grobbelaar *et al*., 2010). A study that assessed the genetic diversity of chicken populations in Africa, Asia and Europe revealed that the Fayoumi breed was grouped with chickens from the Mediterranean, whereas the Koekoek shared a cluster with eastern European breeds and broiler chickens (Lyimo *et al*., 2014). This genetic origin suggests that breeds might respond distinctively in different agro-ecologies. Even though the origin of the breeds was not in Ethiopia, we interpreted its current occupation area as a success in productivity and as evidence for suitability in the current range. Therefore, the current area of occupation could be used to predict suitability for other regions in the country where the breeds are not present.

This novel approach can find practical use in breeding programs, as it can be applied at different scales for different livestock breeds. For region-specific breeds, such as the indigenous Horro chickens (Wondmeneh, 2015), or cosmopolitan breeds, such as the Holstein Friesian cattle, these tools can be useful to predict suitability to a given region, given the climatic variables. The approach can be used when the interest is in designing a breeding plan, introducing a breed to a new area, or when trying to understand differences in performance within the same breed in different areas or between breeds in the same area. To extend the use of prediction models, further analysis can be explored by taking productivity data into account. However, productivity data are difficult to obtain from smallholder farms.

Understanding the environmental requirements of different breeds is an important tool to support higher productivity in particular regions (Arthur and Albers, 2003). As regions can have different environmental conditions, it is imperative to understand how livestock adapt to their environment, and which variables are shaping the differences in performance between breeds.

Breeding programs in developing countries are often ineffective as a consequence of the non-adaptability of the introduced breeds to the challenging environments (Montaldo, 2001; Ojango and Pollott, 2002). More recently Ferreira *et al*. (2017) and Rosé *et al*. (2017) showed that differences between temperate and tropical climates can cause significant genotype by environment interaction (G×E), which affects productivity. This breed-by-environmental mismatch is usually estimated as G×E, the genetic correlation for a given set of traits estimated in two environments. Given the genetic correlation, our methodology can be used to analyze these two environments and predict in which regions a breed will most likely exhibit an environmental mismatch. By analogy it can also reveal potential areas of successful introduction, contributing to a successful breeding program.

In conclusion, this work showed the utility of habitat distribution models applied to a livestock research. This allows making predictions of breed-specific suitability taking into account environmental information. Being able to explain the role of G×E can be a useful application of the methodology developed here, that will further help in providing support when designing breeding programs, or introduction programs for local animal production, by understanding the environmental variables that can have an impact on breed productivity between environments.

## Supplementary material

For supplementary material accompanying this paper visit http://dx.doi.org/10.1017/S1751731118003002. click here to view supplementary material
