# Supplementary material for: Use of geographic information system tools to predict animal breed suitability for different agro-ecological zones
Source: Animal. 2018 Nov 13;13(7):1536–43. doi: 10.1017/S1751731118003002 (PMC6582082; doi:10.1017/S1751731118003002)
Supplement: Supplementary file 1 [file S1751731118003002sup001.docx]

***animal* journal**

**Supplementary material**

**Use of Geographic Information System tools to Predict Animal Breed Suitability for Different Agro-Ecological Zones**

Maria Lozano-Jaramillo^1^*, John WM Bastiaansen^1^, Tadelle Dessie^2^, Hans Komen^1^

^1^*Wageningen University & Research Animal Breeding and Genomics, P. O. Box 338, 6700 AH Wageningen, The Netherlands*

^2^*International Livestock Research Institute, P. O. Box 5689 Addis Ababa, Ethiopia*

*Corresponding author, e-mail: [maria.lozanojaramillo@wur.nl](mailto:maria.lozanojaramillo@wur.nl)

**Supplementary Figure S1.** Characterization of the environmental variables (see Supplementary Table S1 for abbreviations) at the current distribution of each chicken breed.


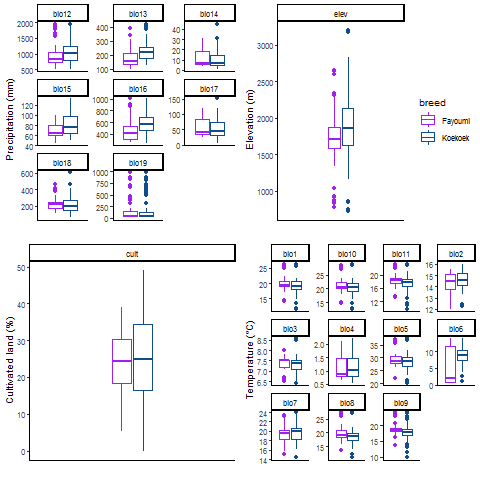


**Supplementary Table S1.** Environmental variables used to build the distribution models.

| **Environmental Variable** | **Description** |
| --- | --- |
| BIO1 | Annual Mean Temperature |
| BIO2 | Mean Diurnal Range (Mean of monthly (max temp - min temp)) |
| BIO3 | Isothermality (BIO2/BIO7) (* 100) |
| BIO4 | Temperature Seasonality (standard deviation *100) |
| BIO5 | Max Temperature of Warmest Month |
| BIO6 | Min Temperature of Coldest Month |
| BIO7 | Temperature Annual Range (BIO5-BIO6) |
| BIO8 | Mean Temperature of Wettest Quarter |
| BIO9 | Mean Temperature of Driest Quarter |
| BIO10 | Mean Temperature of Warmest Quarter |
| BIO11 | Mean Temperature of Coldest Quarter |
| BIO12 | Annual Precipitation |
| BIO13 | Precipitation of Wettest Month |
| BIO14 | Precipitation of Driest Month |
| BIO15 | Precipitation Seasonality (Coefficient of Variation) |
| BIO16 | Precipitation of Wettest Quarter |
| BIO17 | Precipitation of Driest Quarter |
| BIO18 | Precipitation of Warmest Quarter |
| BIO19 | Precipitation of Coldest Quarter |
| ELEV | Elevation |
| CULT | Total cultivated land |
